# Improving Genomic Selection for Heat Tolerance in Dairy Cattle: Current Opportunities and Future Directions

**DOI:** 10.3389/fgene.2022.894067

**Published:** 2022-06-13

**Authors:** Evans K. Cheruiyot, Mekonnen Haile-Mariam, Benjamin G. Cocks, Jennie E. Pryce

**Affiliations:** ^1^ School of Applied Systems Biology, La Trobe University, Bundoora, VIC, Australia; ^2^ Centre for AgriBiosciences, Agriculture Victoria Research, AgriBio, Bundoora, VIC, Australia

**Keywords:** heat tolerance, dairy, Australia, genomic selection, snps, prediction accuracy

## Abstract

Heat tolerance is the ability of an animal to maintain production and reproduction levels under hot and humid conditions and is now a trait of economic relevance in dairy systems worldwide because of an escalating warming climate. The Australian dairy population is one of the excellent study models for enhancing our understanding of the biology of heat tolerance because they are predominantly kept outdoors on pastures where they experience direct effects of weather elements (e.g., solar radiation). In this article, we focus on evidence from recent studies in Australia that leveraged large a dataset [∼40,000 animals with phenotypes and 15 million whole-genome sequence variants] to elucidate the genetic basis of thermal stress as a critical part of the strategy to breed cattle adapted to warmer environments. Genotype-by-environment interaction (i.e., G × E) due to temperature and humidity variation is increasing, meaning animals are becoming less adapted (i.e., more sensitive) to changing environments. There are opportunities to reverse this trend and accelerate adaptation to warming climate by 1) selecting robust or heat-resilient animals and 2) including resilience indicators in breeding goals. Candidate causal variants related to the nervous system and metabolic functions are relevant for heat tolerance and, therefore, key for improving this trait. This could include adding these variants in the custom SNP panels used for routine genomic evaluations or as the basis to design specific agonist or antagonist compounds for lowering core body temperature under heat stress conditions. Indeed, it was encouraging to see that adding prioritized functionally relevant variants into the 50k SNP panel (i.e., the industry panel used for genomic evaluation in Australia) increased the prediction accuracy of heat tolerance by up to 10% units. This gain in accuracy is critical because genetic improvement has a linear relationship with prediction accuracy. Overall, while this article used data mainly from Australia, this could benefit other countries that aim to develop breeding values for heat tolerance, considering that the warming climate is becoming a topical issue worldwide.

## Introduction

Globally, it is projected that milk production needs to double by mid-century to meet the demands of the growing population (Britt et al., 2018). However, rising global temperatures are now becoming a growing issue, affecting humans and threatening further increase in livestock production. In the dairy industry, potential heat stress calculated from temperature and humidity data above specific comfort zones has been increasing worldwide (Silanikove and Koluman, 2015; Polsky and von Keyserlingk, 2017), making this a challenge that compromises production (reduced growth and milk production) and reproduction and sometimes causes death in extreme cases. As such, proactive and multi-pronged measures are needed to prevent current and future economic losses and contribute to feeding the burgeoning population.

Dairy cattle are especially prone to environmental heat stress because they generate high metabolic heat from the fermentation of additional dry matter during lactation. Together, this is becoming a big issue, especially when producers seek to increase production and even more while dealing with the consequences of heat stress at the same time. Conservative estimates show that heat stress alone is currently causing substantial annual economic losses to the dairy industry of up to USD 897 million in the United States (St-Pierre et al., 2003); up to AUD 300 [∼USD 215 million based on February 2022 exchange rate] million in Australia (DairyBio, 2018; https://dairybio.com.au/); and up to £33 million [∼USD 45 million based on February 2022 exchange rate] in the South-West region of the United Kingdom (Fodor et al., 2018).

The abovementioned monetary losses are expected to increase in the coming decades as the climate gets warmer across the world. A recent report by the Intergovernmental Panel on Climate Change (IPCC) indicates that the global temperature has already risen by 1.0°C when compared to the pre-industrial climate and is now projected to increase further in the coming decades all around the world (IPCC, 2021). Global warming of 1.5°C is harmful to humans and agriculture because there will be increased heat waves and long and dry warm seasons (IPCC, 2021). Australia (BOM, 2020) and many other countries, such as the United States and parts of Europe (Polsky and von Keyserlingk, 2017), are already experiencing these devastating climate scenarios, which are now threatening the sustainability of global dairy systems and other sectors.

The literature is rich with reviews on the impacts and approaches to minimize heat stress in livestock and other species, which can be broadly classified into two: management (e.g., use of shade, sprinklers, and ration modification) and genetics (e.g., genomic selection and gene editing) (Renaudeau et al., 2012; Das et al., 2016; Polsky and von Keyserlingk, 2017; Carabaño et al., 2019; Hansen, 2020; Osei-Amponsah et al., 2020). Obviously, the most effective strategy is to implement both management and genetic solutions.

**FIGURE 1:**
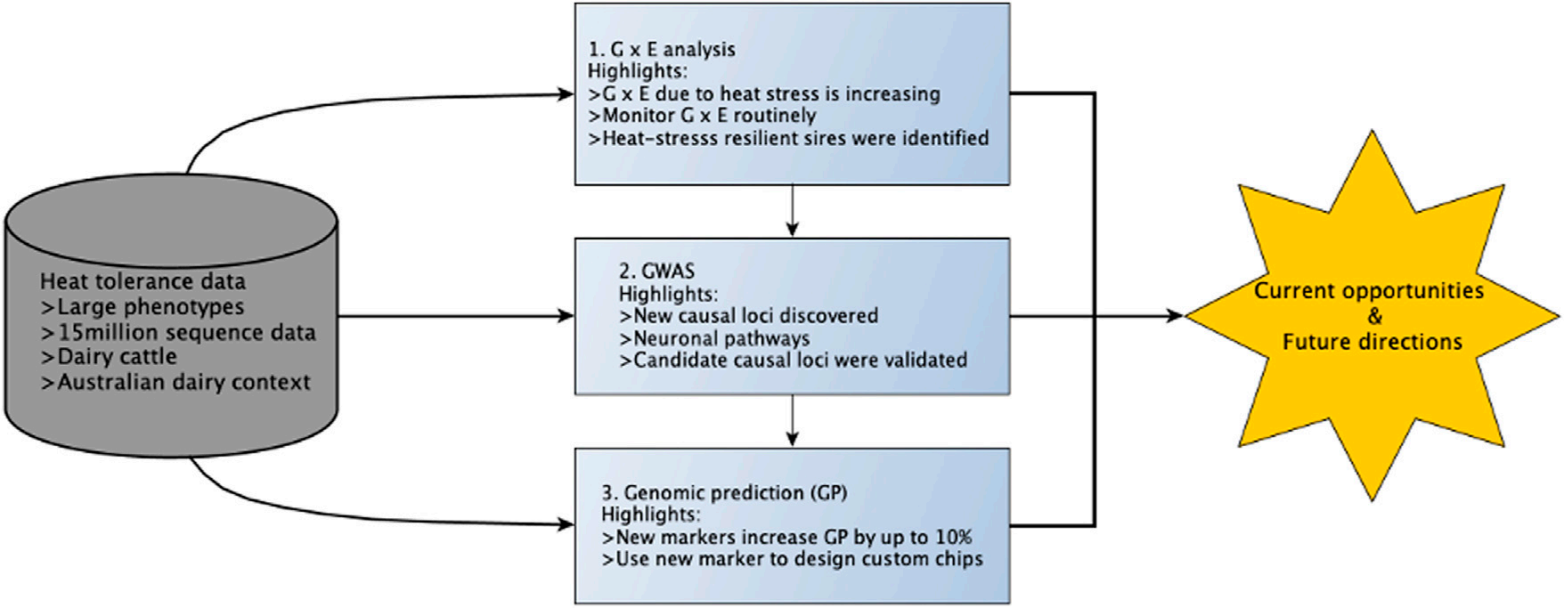
Overview of this review. The boxes colored in light blue represent the three research studies covered in this article and their respective key findings.

Australia is currently at the forefront of using genomics to improve heat tolerance following the development and release of the first breeding values for this trait in 2017 (Nguyen et al., 2016; Nguyen et al., 2017). Overall, there is much interest in understanding the genetic mechanisms that confer thermotolerance in animals, and this is now an active area of research in many countries. In this review, we discuss opportunities and challenges for improving heat tolerance in dairy cattle by drawing on discoveries from recent Australian studies (Cheruiyot et al., 2020; Cheruiyot et al., 2021; Cheruiyot et al., 2022) as an example. These studies were important for several reasons: 1) high-producing dairy cattle (e.g., Holstein) used in these studies are an excellent and convenient model to understand the biology of heat tolerance because they are prone to heat stress from the additional metabolic heat of lactation; 2) the study animals (i.e., Australian dairy cattle) are suited for understanding the genetic basis for which mammals cope with heat stress because they are predominantly managed outdoors on pasture with limited management strategies to alleviate heat stress, in contrast to other countries (e.g., the United States, Canada, and Israel), where dairy cattle are kept indoors and fed total mixed rations (TMR) (Cheruiyot et al., 2020); 3) the dataset size was much larger than those used in earlier comparable studies in dairy cattle (Wang et al., 2017); and 4) these studies used powerful statistical tools to discover genetic variants and test genomic predictions for heat tolerance. In this article, we will first describe indicators of heat tolerance based on milk yield traits and their utility to quantify the genetic difference between animals (Cheruiyot et al., 2020). Second, we will explore how big phenotypic and genomic data can be used to enhance our knowledge of the biology of heat tolerance. Finally, we will discuss pathways for which the industry can benefit from genomic information in a bid to select animals that can maintain productivity under hot and humid conditions and suggest future considerations. An overview is provided in Figure 1.

## Describing Heat Tolerance

Heat tolerance is a complex trait governed by a myriad of biological processes: cellular, morphological, behavioral, and physiological systems [see more information in Sejian et al. (2018)]. This implies that no single measure can completely capture heat tolerance. While physiological measures such as changes in core body temperature are often seen as the “gold standard” proxy for genetic studies of heat tolerance in cattle (Carabaño et al., 2019), it is still too costly and labor-intensive to obtain measures of body temperature on the thousands of animals that would be needed for genomic evaluation, although this situation is rapidly changing with the development of high-throughput sensor technologies in recent years (Koltes et al., 2018).

**FIGURE 2:**
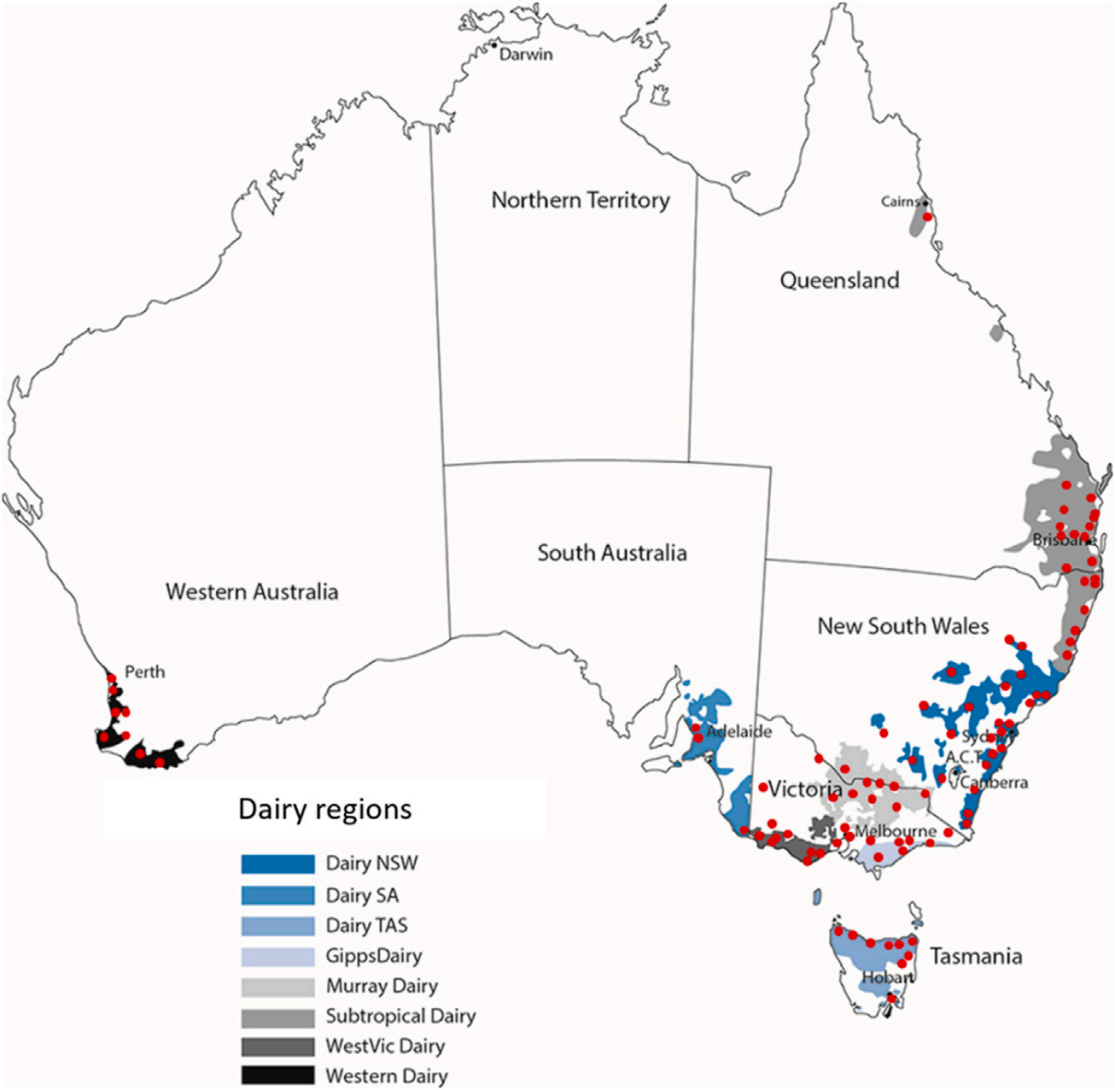
Locations of dairy herds (red points) used in the analysis of heat tolerance.

**FIGURE 3:**
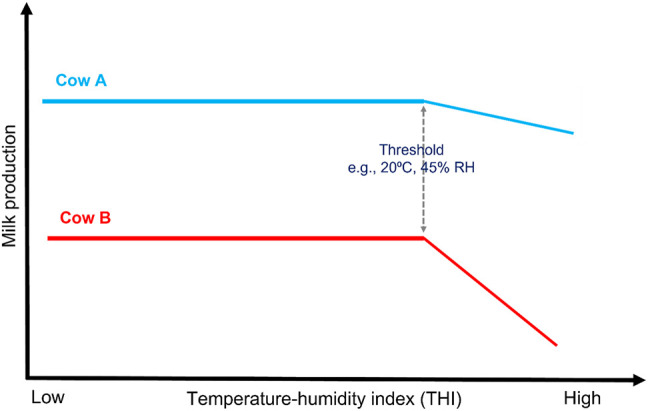
Illustrative description of heat tolerance. Cow A and Cow B produce a comparable quantity of milk at thermoneutral conditions (i.e., at low THI). As the THI increase, the milk yield at first remains unaffected up to a given point called a threshold at which the yield begins to decline for both cows, but the rate of decline (slopes) is more for Cow B than Cow A which is G × E. Therefore, Cow A is considered more tolerant to heat than Cow B, and the slope trait can be for milk, fat, and protein yield.

Besides core body temperature, using alternative measures of heat tolerance based on milk records in dairy cattle began around the year 2000 with the development of novel heat stress models (Ravagnolo et al., 2000). With these models, test-day milk production records are combined with temperature and humidity data from the public weather stations near dairy farms and used to derive a measure of the rate of decline in milk yield associated with an increase in temperature and humidity (slope traits) that can serve as a proxy for heat tolerance. In all the three research studies covered in this review, and as in previous comparable work, for example, in Australia (Nguyen et al., 2016) and the United States (Sigdel et al., 2019), the reaction norm models were used to analyze the combined set of test-day milk records and climate data that were collected across dairying regions in Australia (Figure 2). Comprehensive details of the methods used to derive heat tolerance (i.e., slopes; Figure 3) traits are described in the study by Cheruiyot et al. (2020).

## G × E due to Heat Stress

G × E exists when the relative performance of different genotypes changes across different environments. G × E that results in re-ranking of genotypes is a growing issue worldwide as the production environments continue to become increasingly variable (seasonal and temporal), driven mainly by climate changes. In the Australian context, dairy herds are located mainly in the coastal areas of the country, with a large proportion of herds concentrated in the Victoria region in the South-East part of the country (Figure 3). The average daily ambient temperature varies widely among dairying regions; for example, it ranges from −5 to 38°C in Northern Victoria (Nguyen et al., 2016). Given this regional and temporal diversity in climatic conditions, a relevant question is understanding how big G × E due to heat stress is in Australia? And what are the implications for the industry? In practice, a general guideline is that if a genetic correlation for a trait measured between two environments is below 0.80, then G × E is an issue, and producers could consider forming separate breeding programs to optimize production for each environment (Robertson, 1959).

**FIGURE 4:**
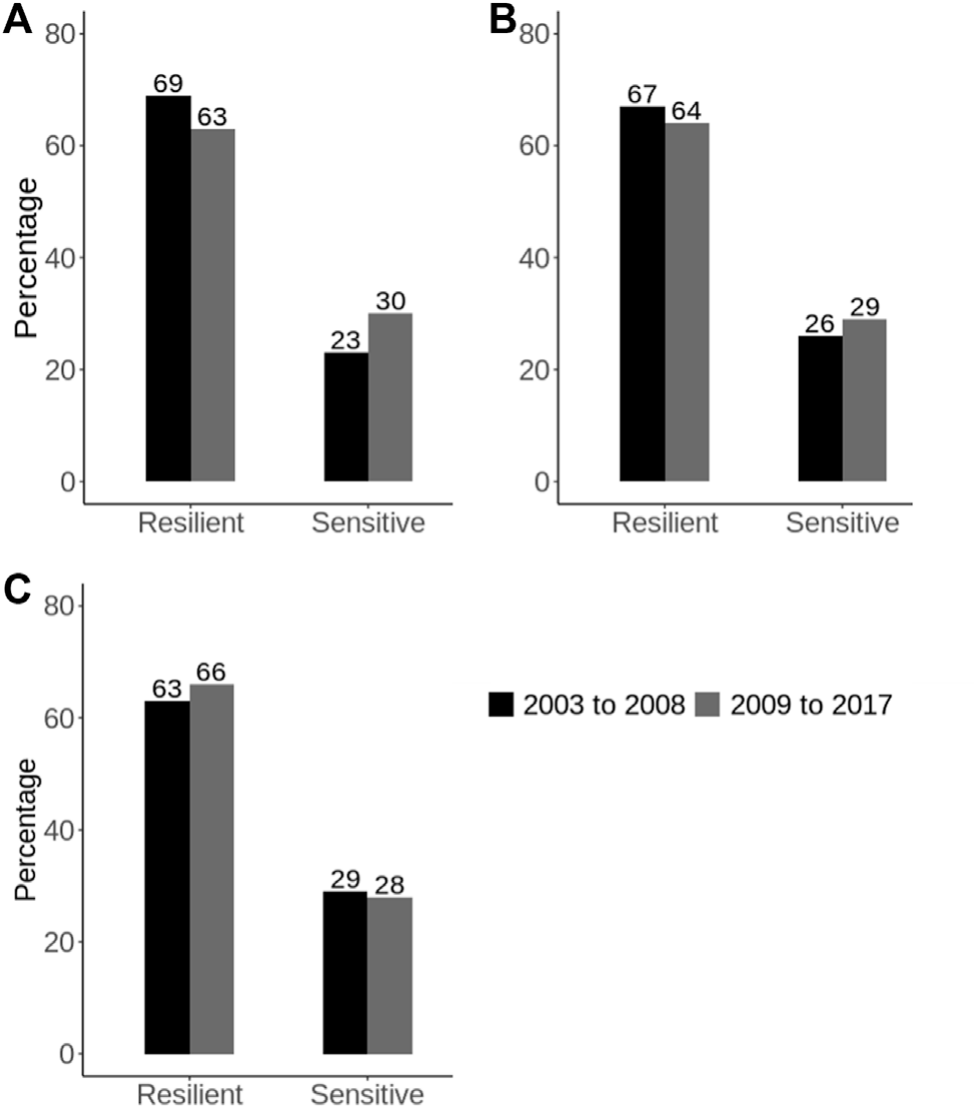
Comparing the percentage of heat stress resilient and sensitive bulls in Australia between earlier years (2003–2008) versus recent (2009–2017) years [Adapted from (Cheruiyot et al., 2020)]. The heat-tolerance profiles of the bulls were defined based on the reaction norms of their EBVs along the THI trajectory for heat tolerance milk **(A)**, fat **(B)**, and protein **(C)** yield slope traits.

Cheruiyot et al. (2020) found that the genetic correlation for heat tolerance traits between the extreme trajectory of THI (i.e., an environmental descriptor of heat stress) was greater than 0.80 across all the study traits meaning that G × E due to heat stress is not likely to be an issue now in Australia. However, results also showed that the G × E due to heat stress has increased over the recent decades in Australia, which agrees with earlier reports (Nguyen et al., 2017). For example, when considering milk yield records, the proportion of sires labeled as heat-sensitive [identified based on the absolute slope values of their reaction norms, as described in the study by Cheruiyot et al. (2020)] has increased by 7% in recent (2009–2017) compared to earlier (2003–2008) years (Figure 4). This is a worrying trend showing that dairy cattle are becoming less adapted to the environments, which can be attributed to two main reasons: 1) increase in climate changes characterized by frequent and extreme weather, and 2) selection pressure on production traits over the years, given that heat tolerance (i.e., slope traits) is unfavorably associated with milk yield (i.e., intercept traits), with phenotypic correlations of around −0.80 (Cheruiyot et al., 2020). This implies that Australian dairy animals are becoming more specialists, which requires more homogeneous and stable environments to maximize performance. Given these results, we recommend that it is important for the industry to routinely monitor this G × E, given that the environments will continue to warm over the coming decades (BOM, 2020; IPCC, 2021). This also applies to other countries around the world that are currently experiencing a warming climate.

If this G × E trend continues, then it would appear that dairy animals are becoming less adapted to the environment, and subsequently, it would be necessary to continuously modify production environments to fit or optimize the productivity of animals. Indeed, this environmental modification is already becoming a common feature in many farming systems worldwide. For example, several adaptation measures are being sought in pig production systems in many countries to minimize the effect of heat stress, including designing special housing with good ventilation, reducing stocking density, and modifying or shifting feeding regimes (Schauberger et al., 2019). Similar adaptation measures are being implemented in dairy industries across the world, including Australia, for example, the Cool Cows program (www.coolcows.com.au), where farmers are provided with the best on-farm practical solutions using shades, sprinklers, or fans to offset heat stress on their farm animals and ensure adequate ventilation for the housed or feedlot cattle.

Innovative studies are currently ongoing in some countries aimed at designing special diets for heat-stressed cows that lower core body temperature and allow them to continue feeding under heat stress conditions, for example, the Feeding Cool Cows program in Australia (https://dairyfeedbase.com.au/feeding-cool-cows/). Moreover, complementary permanent genetic solutions have been recommended to better cope with changing environments, such as the genomic selection of heat tolerance in Australia (Nguyen et al., 2017) or genome editing (Hansen, 2020).

**FIGURE 5:**
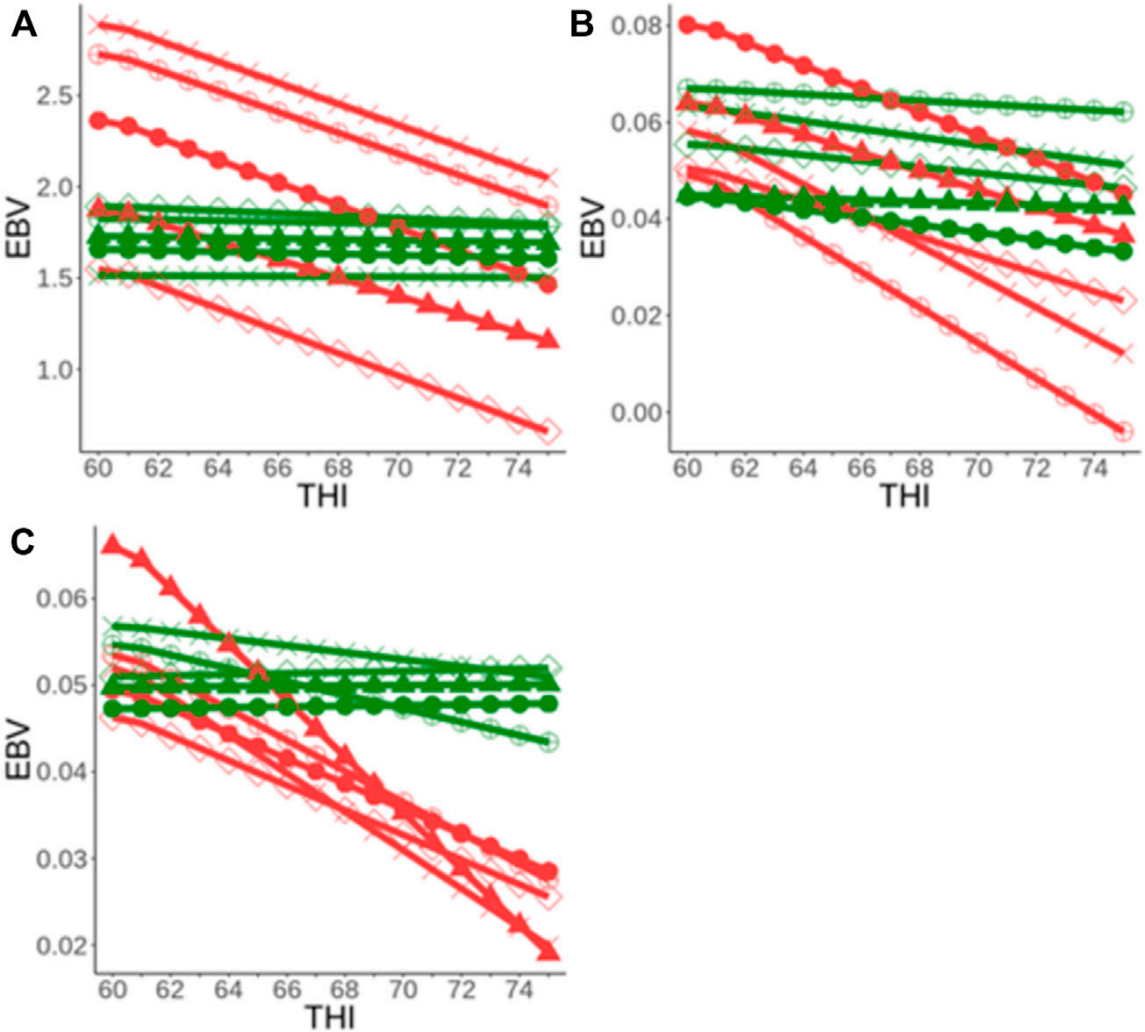
Reaction norm of the EBVs along the trajectory of THI (heat stress) for a sample of bulls in Australia for heat tolerance milk **(A)**, fat **(B)**, and protein **(C)** yield slope traits [adapted from (Cheruiyot et al., 2020)].

In addition, substantial genetic variation for heat tolerance exists among animals (Cheruiyot et al., 2020; Nguyen et al., 2016; Ravagnolo et al., 2000). This is illustrated by the reaction norms of a sample of sires for heat tolerance milk (HTMYslope), fat (HTFYslope), and protein (HTPYslope) traits (Figure 5). Two major groups of sires considered as heat-sensitive (red color) and heat resilient (green color) were identified (Cheruiyot et al., 2020). These results suggest that they are opportunities for farmers to choose heat-resilient (i.e., those that can maintain performance across a wider range of heat stress environments) to optimize production in warmer and variable environments.

## Exploring Resilience Indicators for Heat Stress

**FIGURE 6:**
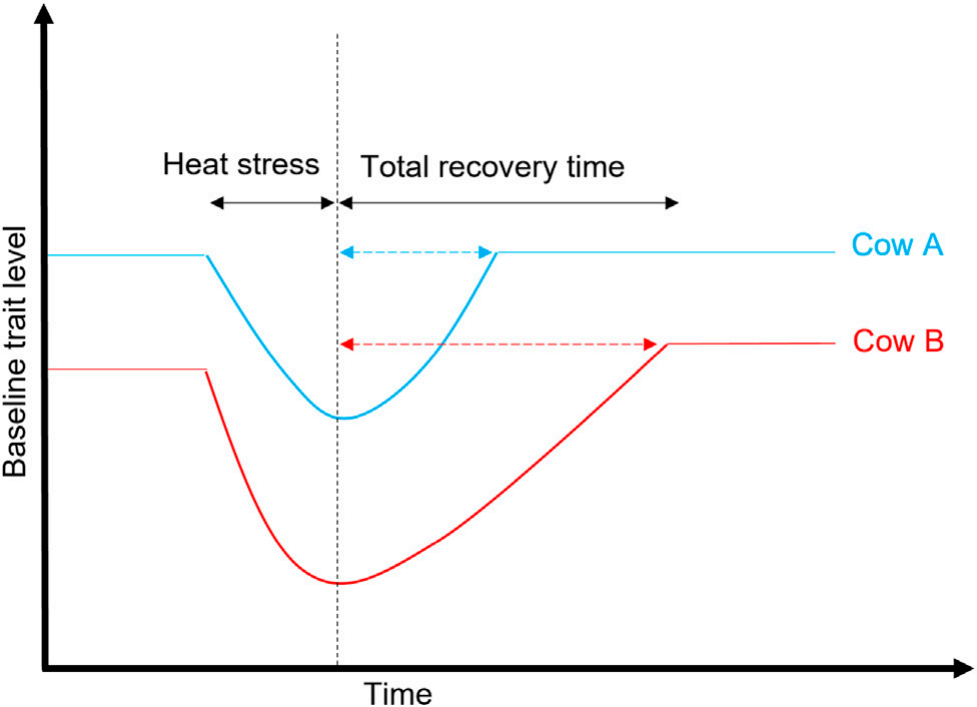
Illustration of heat stress and recovery period between two cows: Cow A is more resilient to heat than Cow B because its trait (e.g., milk yield) returns to the baseline more quickly after exposure to heat stress or other environmental stressors (e.g., disease and parasites).

Resilience is defined as the ability of an animal to recover quickly following exposure to a perturbation(s), or the ability to be minimally affected by a disturbance (Colditz and Hine, 2016), as illustrated in Figure 6. Since G × E due to heat stress is becoming a growing issue in the agricultural sector worldwide, it is relevant to consider resilience indicators in the breeding goals to help accelerate the genetic improvement for this trait and other functional traits (e.g., health and fertility). This is a growing area of research, particularly in developing indicator traits for resilience that encompasses different aspects of animal wellbeing (weather, pathogens, diseases, and social perturbations) (Berghof et al., 2019). This is becoming increasingly possible with the availability of big data sets facilitated by the advancement in phenotyping technologies. A good example of resilience is that the milk yield in heat-tolerant cows (high EBVs for heat tolerance) returned to the baseline level after 6 days following heat challenge in climate chambers compared to nine days in heat-susceptible cows (Garner et al., 2016).

Depending on the trait, resilient animals are expected to have smaller values on the following components compared to the average population: variance (closer to zero), autocorrelation (an indicator of the recovery period as illustrated in Figure 6), skewness of deviations, and slopes (Scheffer et al., 2018; Berghof et al., 2019). Poppe et al. (2020) reported low to moderate genetic correlations among three resilience indicators (variance, autocorrelation, and skewness) that describe deviations of milk yield from a lactation curve, with heritability ranging from 0.01 (skew) to 0.24 (variance). The existence of genetic variation in the recovery period (Garner et al., 2016) means there are opportunities to explore and identify animals that are resilient to environmental perturbations. Although we still do not have a clear understanding of the genetic basis for plasticity to environmental changes, progress has been made to find indicators for resilience (Berghof et al., 2019).

With increasing climate change and variability on top of the increased burden of multiple environmental disturbances (heat, diseases, parasites, nutrition, precipitation, management, and variability in climate), it seems logical to expect the development of a breeding value called “general resilience” in the future, which encompasses multiple traits that contribute to farm profitability, including heat tolerance, and recovery period to different challenges. The top-ranking animals for general resilience breeding value are expected to have smaller phenotypic variance or deviation relative to the average population, and their performance can return to baseline more quickly.

## Exploring Suitable Heat Load Measures for the Dairy Industry

As in previous work in Australia (Hayes et al., 2003; Nguyen et al., 2016), the temperature–humidity index (THI) was used to quantify environmental heat load on the animals and others (Dikmen et al., 2008; Hammami et al., 2015). THI is a single value that combines ambient temperature and humidity. Since this index misses relevant pieces of information which contribute to heat stress in animals, such as wind speed and solar radiation, the magnitude of heat load on animals may have been underestimated in these studies. This is important as dairy cattle in Australia are mostly kept outdoors on pastures where direct solar radiation is also a key risk factor. In the G × E analysis (Cheruiyot et al., 2020), THI = 60 [based on (Yousef, 1985)] was set as the threshold beyond which milk yield in the study animals begins to decline due to heat stress following the work of Hayes et al. (2003. This threshold value may change if a more comprehensive THI is defined in the future. Notably, compared to the commonly used THI variants based on the formulas by National Research Council (1971), THI of 60 (based on Yousef, 1985) is equivalent to THI = 60 [THI = (1.8 × T°C + 32)−(0.55−0.0055 × RH%) × (1.8 × T°C−26)] and THI = 68 [THI = (0.55 × Tdb + 0.2 × Tdp) × 1.8 + 32 + 17.5] [See detailed description of these formulae in Bohmanova et al. (2007)]. It may be desirable to have a unified THI calculation approach to allow comparability of results across studies. However, this is not trivial since specific THI formulae might not be applicable to a wide range of environmental conditions and farming systems.

While THI variants that incorporate solar radiation and wind speed have been tested and shown to work well for housed dairy cattle in subtropical environments (Dikmen and Hansen, 2009), its suitability for cattle kept on pasture has not been tested due to a lack of information (wind speed and solar radiation). However, the development of heat load indices that are better suited for measuring heat load for various production conditions is an ongoing area of research. Wang et al. (2018) used data from two sets of experimental cows [one in housed climate chambers at the University of Arizona, United States, and the other under outdoor conditions at the University of California, Davis, United States] and developed an index called Equivalent Temperature Index for Cattle (ETIC) which combines the effects ambient temperature, relative humidity, wind speed, and solar radiation and their interactions. These authors found that the ETIC outperforms previous THI measures in quantifying heat load on dairy animals under outdoor conditions. There are currently no empirical studies to compare the suitability of THI models versus other models that incorporate solar radiation for assessing heat stress from an Australian dairy perspective and from many other countries. This is necessary to recommend suitable heat stress predictive model(s) for the industry, considering that THI values have been and will continue to be relevant for guiding farm management decisions during hot weather and being crucial for research purposes. In addition, it is critical to define a suitable measure of heat stress, which we will discuss in more detail in the later sections.

## Including Heat Tolerance in the Profit Indices and Potential Challenges

**FIGURE 7:**
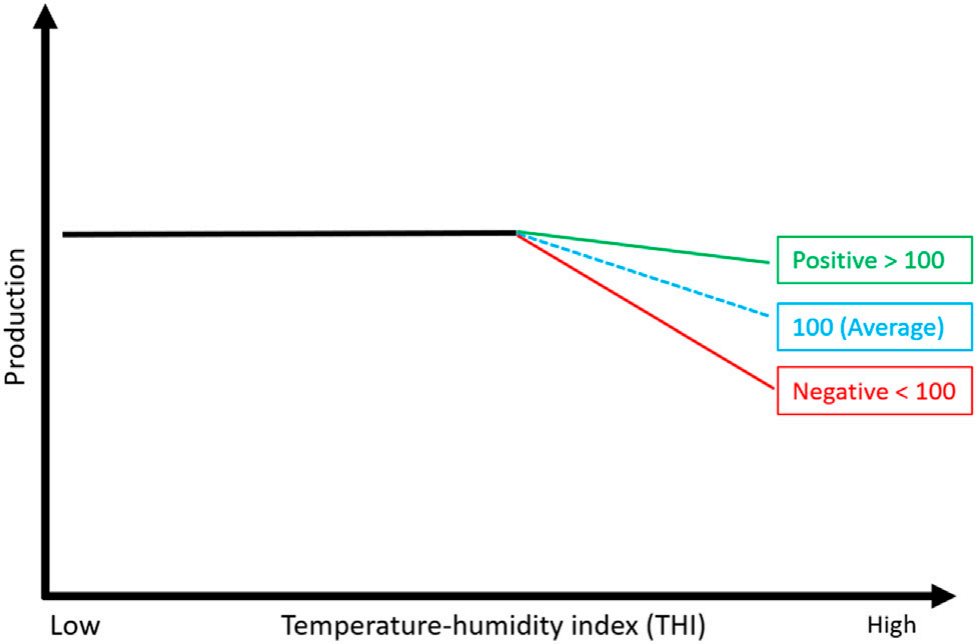
Representation of heat tolerance breeding values (HT GEBVs) that were released to the Australian dairy industry in 2017 (Nguyen et al., 2017). The daughters of a bull with HT GEBV above average (positive) are expected to be more tolerant than the daughters of an average bull and vice versa for bulls with below-average HT GEBVs.

Nowadays, modern dairy cattle are selected on an economic index that optimally combines all traits of economic importance. Australia currently provides two economic selection indices: (Balanced Performance Index (BPI) and Health Weighted Index (HWI), as per farmer preferences for trait improvements (Byrne et al., 2016). These profit indices combine a wide range of traits, such as production, health, fertility, type, and feed efficiency (Byrne et al., 2016), and are calculated using DataGene (https://datagene.com.au/) [an organization responsible for genetic evaluation in the Australian dairy industry]. Notably, heat tolerance is a new trait that has not yet been included in such selection indices worldwide. However, Australia currently provides stand-alone breeding values for this trait to help farmers choose heat-resilient animals. To do this, farmers use a two-step approach to first filter bulls based on BPI and then on heat tolerance. For example, a farmer can look for a bull with a high BPI value, say 350 (meaning this bull is expected to be $350 more profitable than an average bull) and HT-ABV of 105 (meaning this bull is 5% more tolerant to hot/humid conditions than the Australian average score of 100; see Figure 7). Recent data from DataGene show an encouraging upward trend for heat tolerance following the release of HT-ABVs in 2017 (Pryce et al., 2022). However, the uptake of HT-ABVs could be improved by including them directly in the Australian profit index (BPI).

There are a number of challenges that make including heat tolerance into the economic index difficult. First, defining economic weights for use in the selection indices is not a trivial task. However, it is possible to derive economic values for resilience to heat by considering the cost associated with production losses and increased labor costs for managing and treating non-resilient animals (Berghof et al., 2019). Even though it is obvious that resilience has economic merit, the fact that management costs of heat-stressed animals can vary disproportionately among different environments, say in Gippsland (colder climate) versus Queensland (warmer climate) regions of Australia, makes it difficult to standardize economic weights across regions. Similarly, this could be the case in other countries, for example, the United States, where, on average, dairy animals experience greater heat stress in the South than in the North (Key et al., 2014). This variability in heat stress levels across regions is probably one of the major issues that may impede motivation to incorporate heat tolerance in the profit indices (the BPI in Australia). However, one possible way to deal with this issue at this stage could be to put different weights for these traits according to the level of heat stress in a particular region, which could be impractical for national genetic evaluations since it can make extension more complex and confusing for producers. Nonetheless, the fact that climate warming is projected to become an issue across Australia (as discussed earlier) and many regions of the world provides a good reason to act now since multi-trait genetic selection is cumulative and a long-term strategy. This is critical if the goal is to feed the increasing population by the end of the 21st century while at the same time confronting the challenges of warming environments.

## Suitable Traits for Describing Heat Tolerance and for Use as a Breeding Objective

The milk decline traits (i.e., slopes) used as proxies for heat tolerance in some studies covered in this article have been criticized for several reasons: 1) it does not fully capture the effect of heat stress on other economic traits in cattle, 2) it is unfavorably correlated with milk volume with phenotypic correlation estimates of around −0.80 (Cheruiyot et al., 2020), and 3) milk production traits have been already included in the Australian economic indices (Byrne et al., 2016), implying that heat tolerance may have been partly captured in these indices. While this trait (milk decay under heat stress) definition is easier for farmers to understand and somewhat straightforward to include in the profit indices, as described by Nguyen et al. (2017), it is perhaps not the most suitable choice as the breeding objective for heat tolerance, considering the abovementioned reasons.

Alternatively, several other indicators of thermoregulation in animals could be used as the breeding objective, such as those related to core body temperature (e.g., rectal, ruminal, or vaginal temperature), heat production (e.g., feed consumption and fermentation), or latent/sensible heat loss (e.g., skin or cutaneous temperature, sweating rate, and respiratory rate). Among these heat stress indicators, measures of core body temperature, for example, rectal temperature, are often considered as the “gold standard” for heat tolerance with the heritability estimate of 0.17 ± 0.13 (Dikmen et al., 2012) in Holstein cattle. Although this trait could be considered as a breeding objective for heat tolerance, it is questionable by the fact that it is ambiguous as to what it means, for example, to breed for lower rectal temperature (RT), given that the core body temperature in livestock and other mammals are typically tightly controlled within a very narrow range (Finch, 1986; Gourdine et al., 2017). In addition, the genetic correlations between RT and milk production traits are positive (Dikmen et al., 2012; Luo et al., 2021), meaning that selection for lower RT could undesirably impact milk yield. By taking these key factors into account, perhaps a more attractive alternative is to consider traits that capture heat dissipation efficiency, such that high-ranked heat-tolerant animals are defined as those with the superior genetic ability to remove metabolic heat from the core body into the environment. In addition, this will allow continued genetic progress of milk production since high-producing animals can efficiently dispel heat from their core bodies that could have otherwise accumulated due to increased rumen fermentation. This idea was proposed recently by Brito et al. (2020) as one way to breed for heat tolerance and welfare in livestock.

**FIGURE 8:**
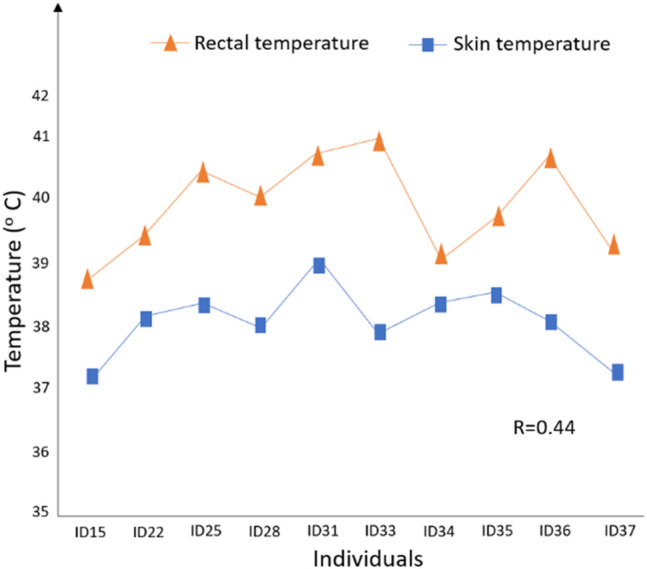
Skin and rectal temperature measures for 10 Holstein calves and correlation (R = 0.44) after heat stress (19:02 h); adapted from Srikanth et al. (2017).

There is evidence that between-breed differences in heat dissipation exist; for example, Finch (1986) observed that *Bos taurus* beef cattle are more superior at dissipating heat than *Bos indicus* beef cattle. Also, within-breed differences in heat transfer exist; for example, Garner et al. (2016) found that the mean skin temperatures of heat-tolerant Holstein cows are significantly higher than those for heat-susceptible Holstein cows. Dikmen et al. (2008) found that slick-haired cattle can control their core body temperature *via* superior thermoregulatory mechanisms compared to non-slick Holsteins, with a relatively lower drop in their milk yield. Srikanth et al. (2017) subjected 10 Holstein calves to heat stress in the experimental chambers and monitored their rectal and skin temperature during the day (Figure 8). These authors found that the rectal temperature for animal ID33 (i.e., one of the study calves) increased rapidly to 41.3°C with relatively lower skin temperature, suggesting that it has poor heat dissipation ability than the other calves (Figure 8). Notably, larger similar studies are required in the future to better discern the relationship between changes in rectal and skin temperature under heat stress conditions. Given that even a small rise in body temperature can have serious negative consequences on cell integrity and metabolic functions, selecting animals that can tightly constrain their core body temperature through superior heat dissipation ability can yield the greatest advantage in productivity (Finch, 1986), including traits such as fertility, and health.

The thermal circulation index (TCI) described by Curtis (1983) quantifies the transfer of heat from the core of the body to the skin surface and then to the environment under steady-state thermal conditions computed as follows: TCI = (T_s_ – T_a_)/(RT – T_s_), where T_s_ is the average skin temperature, RT is the core or rectal temperature, and T_a_ is the ambient temperature. This TCI trait could be used as a proxy for heat dissipation efficiency through the skin, which accounts for up to 85% of total heat loss (Maia and Loureiro, 2005). In addition to T_a_ (ambient temperature), it may be more suitable to calculate TCI based on ETIC (Wang et al., 2018), which (as discussed earlier) combines key weather elements, including ambient temperature, relative humidity, solar radiation, and wind speed and is, perhaps, a better predictor (compared to THI) of the environmental heat load on the animals, especially for the pasture-based system in Australia. Looking at Figure 8, calf ID33 and ID36 are expected to have smaller TCI values because of the relatively high RT and lower T_s_, meaning that they are more susceptible to heat stress and may not be an ideal candidate for hotter environments. Using the TCI trait as the selection target for heat tolerance and welfare could be attractive to farmers because it is likely to drive genetic improvement for this trait, with a possible small impact on production traits, although there is a need to confirm this hypothesis. It may be necessary to standardize TCI so that breeds with inherently lower or higher rectal temperatures have comparable values. Alternatively, the rate of change in body temperature from the comfort threshold due to an increase in ambient temperature can be used as a relevant selection target for heat tolerance. In this case, heat-tolerant animals are expected to have a smaller rate of change than heat-susceptible animals. Future work should explore the suitability of these thermoregulatory traits as potential targets for heat tolerance in animals.

As it is, the TCI and other thermoregulation traits seem to describe the biology of heat tolerance (i.e., heat dissipation efficiency) which, according to the producer’s perspective, may not be an economically appealing target. However, given that producers seek to maintain production and reproduction under heat stress, the economic value for this trait (TCI) can be defined as the loss of milk production associated with the unit increase in core body temperature of the animal, such that animals with large TCI values are expected to experience greater decline in milk production or fertility under heat stress conditions. This implies that future work is needed to estimate the rate of milk decline and fertility associated with an increase in core body temperature or TCI values related to the failure or inefficient thermoregulation. Nevertheless, we still expect lingering doubts on the suitability of TCI as the target breeding objective for heat tolerance over, say, milk decays (i.e., milk slopes used in this study), in part, because of the high cost of obtaining RT and T_s_ measurements at this stage. However, as high-throughput sensor technologies continue to improve (Koltes et al., 2018), which can facilitate the collection of large thermotolerance phenotypes at potentially lower cost, we expect the TCI trait to be a relevant target for heat tolerance in the future because it is likely to best describe heat-tolerance (Finch, 1986). This is intuitive since it can allow simultaneous improvement of heat tolerance and milk production as well as fertility—a critical goal for producers.

## Selection Criteria for Heat Tolerance

The accuracy of the genomic prediction relies on several factors, including 1) the heritability of a trait, 2) effective population size, 3) size of the reference population, 4) marker density, and 5) the architecture of the trait (Daetwyler et al., 2008; Meuwissen, 2009; Hayes et al., 2010; Gonzalez-Recio et al., 2014). With low heritability estimates for thermoregulation traits, for example, the rectal temperature [*h* ^*2*^ = 0.17 in Holsteins; Dikmen et al. (2012)]—a component needed to calculate TCI (described above)—over 20,000 animals of a female reference set would be required to achieve moderate genomic reliabilities of ∼0.40 (Gonzalez-Recio et al., 2014). Real-time time large measurements of these phenotypes in large quantities (i.e., rectal and skin temperature) can be achieved through innovations in sensor technologies (Koltes et al., 2018). While we have seen a rapid evolution in high-throughput sensor technologies in recent years (Koltes et al., 2018), it is still costly and logistically challenging to build a sufficient reference population with phenotypic measurements (i.e., rectal and skin temperature) needed to compute animal TCI (i.e., breeding of objective trait as proposed earlier) values for genomic evaluations.

However, as phenotyping technologies continue to improve and are validated for commercial use (Koltes et al., 2018), an alternative approach at this stage for the dairy industry is to have a dedicated genomic reference population with high-quality phenotypes (i.e., RT and T_s_). Then, other predictor traits which can be collected cost-effectively in large quantities are used as the selection criteria for heat tolerance. For example, clinical mastitis is a hard-to-measure trait where somatic cell count (SCC) and other traits (e.g., udder depth) have been used as indicator traits for the selection of this economically important trait since large datasets for SCC can be obtained from routine milk recordings (Martin et al., 2018). Considering that TCI is a novel trait proposed here as the breeding objective for heat tolerance, a lot of future work is required, such as understanding the trait, gathering enough data, calculating genetic parameters (e.g., heritability), and correlations with other economic traits (production, fertility, and health trait). It will be interesting to find out if there is some level of genetic and phenotypic correlation between milk yield slope and TCI with an objective of developing a multi-trait heat tolerance breeding value.

Opportunities are emerging to obtain potentially inexpensive large number of phenotypic measures from mid-infrared (MIR) predicted milk biomarkers as the proxy for heat tolerance. Hammami et al. (2015) found that the MIR-predicted traits (e.g., C18:1 *cis*-9) decline with increasing THI in heat-stressed cows. van den Berg et al. (2021) found high genetic correlations (values close to 1.0) between measured serum urea and milk MIR-predicted serum urea. This high genetic correlation means that MIR-predicted urea can be used to improve the accuracy of genomic prediction of serum urea. Heat stress increases milk urea in dairy cows, possibly due to elevated deamination of amino acids, increased metabolism of muscle tissues, or reduced feed intake that often occurs under hot weather (Cowley et al., 2015; Gao et al., 2017). Therefore, using phenotypes such as MIR-predicted C18:1 *cis*-9 [which was found to be most sensitive to heat stress by Hammami et al. (2015)] could be used as an inexpensive selection criterion for heat tolerance or used directly to quantify heat tolerance. Alternatively, and as dairy operations continue to be increasingly automated over the past years, the industry can take advantage of the availability of daily milk recordings to gain better insights into the biology of heat tolerance. This can be achieved through a targeted sampling of dairy farms that continuously capture daily milk recordings using tools such as automated milk samples and weather data.

## Understanding the Biology of Thermal Stress: Study Traits, Animals, and Practical Application

### Genome-Wide Association Studies for Heat Tolerance

Characterizing causal variants and pathways underpinning the genetic basis for heat tolerance in cattle is at the infancy stage, but it is gaining increased attention due to global warming, as demonstrated by a recent surge in published studies (Hayes et al., 2009; Dikmen et al., 2013; Macciotta et al., 2017; Luo et al., 2018; Sigdel et al., 2019). In addition, big genomic datasets are increasingly becoming available thanks to international consortiums, such as the 1000 Bull Genomes Project, which currently hosts whole-genome sequence data for cattle from over 40 collaborators around the world (Hayes and Daetwyler, 2019). As discussed earlier, the G × E work by Cheruiyot et al. (2020) clearly showed a substantial genetic variation for heat tolerance in Australian dairy cattle. As such, these researchers did a follow-up GWAS using imputed whole-genome sequence data with the goal to discover the genetic variants that make dairy animals vary in heat tolerance using a large sample size (∼30,000 cows with heat tolerance phenotypes and genotypes).

Similar to other studies, for example, the study by Hayes et al. (2009) in Australia and Sigdel et al. (2019) in the United States, the heat-tolerance phenotypes used to search for heat tolerance variants in the study by Cheruiyot et al. (2021) were derived from milk records (milk, fat, and proteins). These heat tolerance traits are defined as the rate of decline in milk yield traits (slopes) with an increase in THI. However, using slope traits from milk records presents a challenging task in disentangling genes for heat tolerance and milk production because these traits are highly correlated [phenotypic correlation estimates of about −0.80; (Cheruiyot et al., 2020);], implying that they are largely regulated by the same genes. Nevertheless, it is possible to obtain heat tolerance phenotypes that are independent of milk production by decomposing their correlations using approaches such as the principal component analysis (PCA) (Carabaño et al., 2014; Macciotta et al., 2017). Using these traits in association studies can probably allow better insights into the biology of heat tolerance.

It is not surprising to see that studies that used milk decays to search for genes (Sigdel et al., 2019; Cheruiyot et al., 2021) found strong GWAS signals (i.e., QTLs) for heat tolerance overlapping with well-known genes for milk production (e.g., DGAT1), with candidate variants showing opposing effect direction between these traits. These findings suggest that selecting heat tolerance genes could negatively impact milk production. Cheruiyot et al. (2021) performed a follow-up post-GWAS conditional analysis, which confirmed that the overlapping QTLs are important for both heat tolerance and milk production. These findings have implications for breeding, considering that farmers would like to simultaneously improve heat tolerance while at the same time increasing milk production—a key driver for farm profitability. However, experience in the genetic selection of dairy cattle shows that it is possible to improve several economic traits at the same time, even if there is genetic antagonism between them (García-Ruiz et al., 2016).

### Use of Heat Tolerant Genetics and Crossbreeding (Heterosis)

Alternatively, it is possible to achieve simultaneous benefits for heat tolerance and production by taking advantage of breed complementarity and heterosis—defined as the increase in the phenotypic performance of crossbred progeny (F1 generation cross) relative to that of its purebred parents (Bourdon and Bourbon, 2000). Heterosis or hybrid vigor has been largely used to improve economic traits in beef breeds and crops, but there is an emerging trend toward adopting this strategy in dairy cattle. Experiments in beef cattle showed that Hereford × Boran or Hereford × Braham or Tuli crossbreds have superior heat tolerance ability than Hereford cattle under heat stress conditions in climatic chambers (Gaughan et al., 1999). In this regard, future international collaborations may open promising avenues to understand why some breeds excel in a specific trait or to compare and pinpoint specific genetic variants that make adapted breeds (say those from warm climates, such as Zebu) differ from temperate breeds (Holstein) in their thermotolerance.

So far, many GWAS studies aimed at understanding the biology of thermal stress have been focused on Holsteins (Macciotta et al., 2008; Dikmen et al., 2013; Sigdel et al., 2019; Cheruiyot et al., 2021) with little or no information on other breeds such as Jersey, in part, due to the large amount of data for Holsteins. Some evidence suggests that the superior heat tolerance ability of Jerseys is based on their less reduction in milk yield compared to Holsteins (Bryant et al., 2007; Smith et al., 2013). Similar studies (Mylostyvyi et al., 2021) have reported greater heat tolerance ability of Brown Swiss compared to Holsteins. Otto et al. (2019) explored the genetic basis of heat tolerance using crossbred cows (*Bos indicus* vs. *Bos taurus* cattle). Apart from GWAS, another way to unravel the genetic mechanisms that make animals differ in heat tolerance is to use selection signature tools [Fst (Weir and Cockerham, 1984)]. For example, Freitas et al. (2021) explored signatures of selection in different animal populations (*Bos taurus*, *Bos grunniens*, and *Bos javanicus*) and identified genomic regions associated with thermal stress, including heat shock proteins. Similarly, Taye et al. (2017) compared the genome of indigenous African cattle breeds (including Ankole and Boran) and commercial dairy breeds (including Holstein and Jersey) and reported a positive selection of genes associated with morphology (skin characteristics) and biochemical mechanisms (oxidative stress) for heat tolerance in African breeds.

### Genes Underlying Metabolic Adaptations and Other Pathways for Heat Tolerance

Selection for heat tolerance could inevitably impact genetic progress in milk production (Carabaño et al., 2019). This is because the reduction in milk production of high-yielding animals under heat stress conditions is a survival strategy aimed at lowering core body temperature. The findings by Cheruiyot et al. (2021) provide several biological insights into thermal stress that can be leveraged to minimize heat stress while potentially maintaining productivity in high-yielding dairy cattle. For example, metabolic adaptations are key biological mechanisms for heat tolerance. In high-yielding dairy cattle, such as Holsteins breeds, elevated metabolic heat is a major proteotoxic stress that impacts milk production with reductions of up to 40% (Kadzere et al., 2002; West, 2003). As such, understanding the genetic basis underlying metabolic adaptations could allow breeding for heat tolerance while maintaining high productivity.

Several promising candidate genes for heat tolerance identified by Cheruiyot et al. (2021) (*ACLY*, *PDHA2*, *MDH1*, *SUCLG2*, and *PCK1*) are associated with the citrate (Krebs) cycle, which is a crucial metabolic hub in the oxidation of carbohydrates and fatty acids (Belhadj Slimen et al., 2016). Also, Garner et al. (2020) reported many candidate genes (including *BDKRB1* and *SNORA19*) that are differentially expressed under heat stress and thermoneutral conditions associated with thermoregulation, metabolism, and inflammation. These findings are not surprising considering that heat stress disturbs the metabolism of carbohydrates—a major source of energy for maintenance and production in animals, for two main reasons: 1) reduced dry matter intake and 2) altered post-absorptive metabolism (Wheelock et al., 2010). Although studies are still conflicting, some reports show that fatty acids are not mobilized under heat stress, as evidenced by unaltered basal NEFA (associated with negative energy balance) in heat-stressed cows (Rhoads et al., 2010; Wheelock et al., 2010). This is partly related to the inability of heat-stressed cows to employ the “glucose sparing” effect, such that the adipose tissue is not mobilized to generate NEFA, which, in part, explains drastic milk decline (Rhoads et al., 2010; Baumgard and Rhoads Jr, 2013). In contrast, the “glucose sparing” effect is often enlisted in early lactation (i.e., a stage of high energy demand resulting in negative energy balance) to maintain milk production in dairy cows (Baumgard and Rhoads Jr, 2013).

Moreover, a transcriptomic study on mammary tissue (Gao et al., 2019) found that heat stress suppresses the expression of many genes related to metabolic and immune functions. Recent work in China reported a significant difference in the metabolic profile (maltose, glycerol, and mannitol) associated with carbohydrate metabolism between heat tolerant and heat susceptible cows. Also, these researchers found that the rumen microbial composition is altered differently in heat-susceptible versus heat-tolerant cows under heat stress conditions. As a potential marker for heat tolerance, Liu et al. (2017) found that the lipid component (lysophosphatidylcholine) is significantly reduced in heat-susceptible than heat-tolerant cows under experimental heat challenges. Yue et al. (2020) reported eight and 12 metabolites that were altered by heat stress in milk and plasma of Holstein cows. While many potential heat-tolerant diagnostic metabolites have been documented, there is still no consensus on which metabolites are most suitable for describing heat tolerance.

Milk production in dairy cattle and other mammals depends largely on the number and efficacy of the synthetic capacity of the mammary gland epithelial cells. Exposure of dairy cows to heat stress before and during lactation negatively affects the mammary gland epithelial cell development, physiology, and integrity impacting milk production. Dado-Senn et al. (2018) conducted RNA-Seq analysis and identified over 3,000 candidate genes and pathways involved in mammary gland development under heat stress, including upregulation of cell death, cytoskeleton degradation, and immune response. Recent data show that heat stress, even in dry and pregnant cows, can have undesirable carry-over effects on the lifetime performance of their progeny (Laporta et al., 2020), implying severe economic costs to the dairy industry.

### Selecting for Genes Underlying Metabolism and Other Pathways

A fundamental question is whether selecting for the candidate genes (linked with, say, metabolism or mammary development) would contribute to heat tolerance without substantially reducing milk production. This remains an open question because it is not clearly understood how these genes are regulated under acute and chronic heat stress conditions to impact animal productivity. Compared to acute stress, the genetic aspects of chronic heat stress are still poorly understood. Recent research in camel somatic cells suggests that acute and chronic heat stress are somewhat controlled differently with the former associated with the increased heat shock proteins and DNA repair enzymes, while the latter heat-response mechanism is linked to altered cell architecture, proteomics, and cytoskeletal proteins (Saadeldin et al., 2020). Therefore, we think that the candidate genes and pathways discovered by Cheruiyot et al. (2021) provide interesting insights into the genetic basis for chronic (long time heat exposure) or recurrent heat stress—a characteristic of Australian seasonal summers. Future work is needed to confirm this. Notably, the candidate causal genes discussed by Cheruiyot et al. (2021) did not overlap with those reported in previous comparable work in Australia (Hayes et al., 2009; Wang et al., 2017) and in the United States (Sigdel et al., 2019), most likely because they used smaller datasets [typically <5,000 animals, while around 30,000 was used by Cheruiyot et al. (2021)] and low-resolution SNP sets [50k or 600 SNP set versus 15 million SNPs used by Cheruiyot et al. (2021)]. Importantly, the candidate causal variants were confirmed to be relevant for heat tolerance in an independent validation set *via* genomic prediction (to be discussed in later sections). Moreover, the conditional analyses by Cheruiyot et al. (2021) confirmed that the top GWAS hits/signals are associated with the biology of thermal stress in dairy cattle. However, to what extent these variants will be replicated in other independent studies remain to be established.

Under hot conditions, heat-stressed dairy cows employ various behavioral strategies to regulate internal metabolic heat production, such as lowering feed intake, spending less time grazing and more time standing, resting in the shade, and drinking more water (Kadzere et al., 2002). While these behavioral and physiological adjustments imply that milk decline in heat-stressed dairy cows is inevitable, the genetic tools provide opportunities to minimize such losses to the lowest possible level. For example, finding alternative ways that help dairy cows minimize the accumulation of toxic reactive oxygen species (ROS) and the onset of metabolic heat, especially in warmer months, and in an energy-efficient way is critical to breeding heat-tolerant yet high-yielding animals. Heat stress causes an overproduction of ROS, which can cause oxidative stress and subsequent apoptosis or cell death (Belhadj Slimen et al., 2016). There is evidence that efficient scavenging of toxic ROS from cellular metabolism during various environmental stresses is a signature of increased adaptation in plants (Sharma et al., 2012).

In this regard, exploring the appropriate nutrition (e.g., Feeding Cool Cows program in Australia; covered earlier) to suppress core body temperature or those that favor lower heat increment during hot weather is gaining increased attention. However, genetic aspects to achieve these goals (lower ROS, core body temperature, or heat increment) in heat-stressed animals remains largely unexplored. A recent study (Atta et al., 2020) suggests that supplementing heat-stressed rats with Pycnogenol (generic French pine bark extract) stimulates genes related to antioxidant activity allowing them to reverse heat-induced ROS damage in testicular and brain tissues. It will be illuminating to understand the role of metabolic-related genes discovered in Cheruiyot et al. (2021) and other studies (Garner et al., 2020) to breed more productive and heat-tolerant animals. Moreover, it may help in devising novel ways for improving the nutritional management of heat-stressed animals.

### Genes Underlying Neuronal System and Their Applications

The nervous system plays the most critical function in controlling body temperature by connecting the internal and external environment of animals (Nakamura and Morrison, 2008). Cheruiyot et al. (2021) discovered specific candidate causal mutations underlying heat tolerance that implicate nervous system mechanisms (the neuroactive ligand-receptor interaction and glutamatergic synapse gene categories were overrepresented). Interestingly, the most promising genes in the neuronal pathways (*NPFFR2*, *ITPR1*, *ITPR2*, and *GRIA4*) could be relevant for feeding and metabolic homeostasis in cattle during thermal stress, which are new discoveries that may help manage and breed heat-tolerant animals. The genes are linked to neuroendocrine functions and are involved in a cascade of hormonal responses such as secretion of growth hormone, insulin, serotonin, prolactin, adrenaline, renin and thyroxine, and corticosteroids associated with milk synthesis (Bernabucci et al., 2010; Rhoads et al., 2010). Altering the activity of these hormones has consequences on feed intake and metabolism, which in turn impacts milk yield (Rhoads et al., 2010). Transcriptomic studies in avian species (Kim et al., 2017) have also found that genes related to the neuronal system are relevant in response to heat stress.

Since depressed feed intake is a major contributor to milk decline in heat-stressed cows, it would be interesting to see if manipulating candidate genes identified in the neuronal pathways (*NPFFR2* gene) induces hypothermia and stimulates feed intake. For example, recent work by Laible et al. (2021) attempted to lighten the skin color of Holstein through gene-editing in a bid to minimize the absorption of solar radiation during heat stress. Also, research in mice by Zhang et al. (2018) suggests that the deletion of the *NPFFR2* gene results in impaired diet-induced thermogenesis and energy metabolism. Future in-depth interrogations of these genes, such as transcriptomic and biochemical profiles under different environmental conditions, are warranted. Ultimately, this may provoke innovative ways for managing thermal stress, such as designing specific agonist or antagonist compounds that can be used as feed supplements for dairy animals during heat stress conditions. For example, ractopamine and zilpaterol β-agonists, used to enhance weight gain and feed efficiency in livestock, have been developed and approved for commercial use as feed additives in some countries, such as the United States, Canada, and Japan (Centner et al., 2014; Niño et al., 2017).

If similar compounds for minimizing heat stress are developed in the future, one possible way to test their benefits is to compare them with other known naturally occurring compounds with opposite effects on thermoregulation (those promoting heat stress). An excellent example is ergovaline—an ergot alkaloid often found in endophyte-infected species (ryegrass and tall fescue), which is toxic to cattle due to its dopaminergic effects causing disturbances on animal physiology. A number of studies have found an association between ergovaline and predisposition to heat stress in livestock characterized by increased core body temperature, excessive panting, shade seeking (since ergovaline promotes vasoconstriction thus limiting heat dissipation), decreased feed intake, weight gain, and milk production (Klotz and Nicol, 2016). In fact, “fescue toxicosis” is a big issue in the United States, compromising feed intake and productivity in cattle and sheep during summer seasons when animals are fed endophyte-contaminated diets (Paterson et al., 1995; Klotz and Nicol, 2016). We believe that the development of compounds for use in reducing heat stress in livestock is less likely to face significant regulatory hurdles because they can help to improve animal welfare (health problems, hunger, thirst, frustration, and aggression), which is becoming a growing issue worldwide due to global warming. Indeed, a recent survey in Brazil (Yunes et al., 2021) found that the public was more receptive to gene-editing aimed to minimize heat stress than gene-editing geared toward improving muscle growth in animals.

### Selecting for Genes Associated With Heat Dissipation

It is also worthwhile exploring other genetic features that allow dairy cows to better regulate or dissipate heat efficiently under hot weather, such as those related to morphology (coat color, coat length, and hair thickness) and physiological (cardiovascular and respiratory systems) and cellular (cell repair, fluidity, and stability) functions. For example, research has shown that Holstein cows with SLICK hair coats are more efficient at regulating core body temperature with a lower decline in milk yield than wild-type cows under heat stress conditions (Dikmen et al., 2008). Causal mutations for the SLICK phenotype have been mapped to PRLR (prolactin receptor) gene in chromosome 20 at ∼39 Mb in the Senepol cattle (Olson et al., 2003; Littlejohn et al., 2014). As expected, the work of Cheruiyot et al. (2021) did not detect any significant SNP (*p* < 1E-05) near or within the PRLR gene across all their GWAS analyses, likely because the study population (Holsteins) lacks the causal mutation for the SLICK hair phenotype. However, future efforts should aim at introgressing the causal mutation for this gene in the dairy population lacking the SLICK gene to better cope with heat stress, such as those implemented for Holstein cattle in Puerto Rico and the United States (Davis et al., 2017; Hansen, 2020).

So far, Holstein cows that are primarily heterozygous for the SLICK genotype have been confirmed to possess superior heat tolerance ability over wild-type cows (Dikmen et al., 2008; Dikmen et al., 2014). However, a crossbreeding program was initiated in New Zealand to build homozygous SLICK bulls with up to 75% New Zealand dairy genetic background (Davis et al., 2017). On top of these efforts, it would be interesting to see if additional benefits could be achieved when breeding for homozygous SLICK bulls + high HT-ABV (i.e., HT-ABV + SLICK genotype). As discussed earlier, the heat tolerance capacity of Australian dairy cattle is ranked based on genomic estimated breeding values (HT-ABV), such that animals ranked high for HT-ABV are considered more tolerant to heat than the average population (Figure 7). Top ranking cows for HT-ABV were found to maintain lower core body temperature and experience less milk yield decline under heat stress, which is thought to be related to their efficient heat dissipation mechanisms and energy metabolism (Garner et al., 2016). Overall, we expect more benefits from a breeding program that aims to build dairy bulls with high HT-ABV values and carry homozygous SLICK genotype. However, extensive performance data is needed to confirm this hypothesis.

### Using Prioritized Sequence Variants to Drive the Genetic Gain for Heat Tolerance

It is not enough to merely discover candidate variants controlling heat tolerance without demonstrating their relevance in animal breeding and other species. As such, Cheruiyot et al. (2022) tested if adding sets of prioritized sequence variants into the standard-industry 50k SNP array enhances the prediction accuracy of heat tolerance in dairy cattle. This is relevant because the genetic improvement for a trait is linearly related to the accuracy of estimated breeding values (EBVs), selection intensity, and genetic variation and is inversely proportional to the generation cycle (Schaeffer, 2006). Even a smaller lift in prediction accuracy is valuable to the wider industry with respect to the economic efficiency of breeding programs.

The work by Cheruiyot et al. (2022) found an increase in accuracy of up to 10% in some scenarios when the pre-selected set of sequence markers (∼9,000 SNPs) for heat tolerance were added to the standard-industry 50k SNP panel. However, the changes in prediction accuracies (i.e., with or without using pre-selected markers) varied considerably depending on the scenario tested. These results are encouraging, showing that the selected sequence variants are functionally relevant for heat tolerance and can be used to drive the genetic gain for this trait. This knowledge can benefit the dairy industry in tackling the challenges of heat stress in three practical ways: 1) including them in the standard 50k SNP arrays used for routine genomic evaluations (Cheruiyot et al., 2022), 2) using these variants to design customized SNP panels (Xiang et al. (2021), and 3) leverage recent sequencing technologies (skim sequencing) to cost-effectively genotype pre-selected SNPs for use in screening (Martin et al., 2021; Snelling et al., 2020) heat tolerance genotypes.

### Incorporating Prioritized SNPs for Heat Tolerance in the Industry Custom Panels

As a case scenario, the Australian dairy industry currently uses the 50 k SNP array in routine genomic evaluations implemented by DataGene [(https://datagene.com.au/); Melbourne, Australia]. This organization receives genotypes and animal information from various authorized genomic service providers (GSPs). Usually, these GSPs supply genotypes for animals from various lower density SNP chips (only those accepted by DataGene), which are then imputed to the standard 50k SNP array. To do this, DataGene has a special reference set of animals for imputation with real standard 50k genotypes. Building and optimizing such robust reference set and SNP chips (50k) requires considerable work and resources, including SNP discovery, filtering high-quality SNPs (variants with high polymorphism, MAF, and call rate), and deploying onto a genotyping assay. This implies that while functionally relevant variants for heat tolerance were discovered and validated by Cheruiyot et al. (2022), we should expect a lag of time before an “imputation reference” set of animals is assembled with real genotypes for these variants.

**FIGURE 9:**
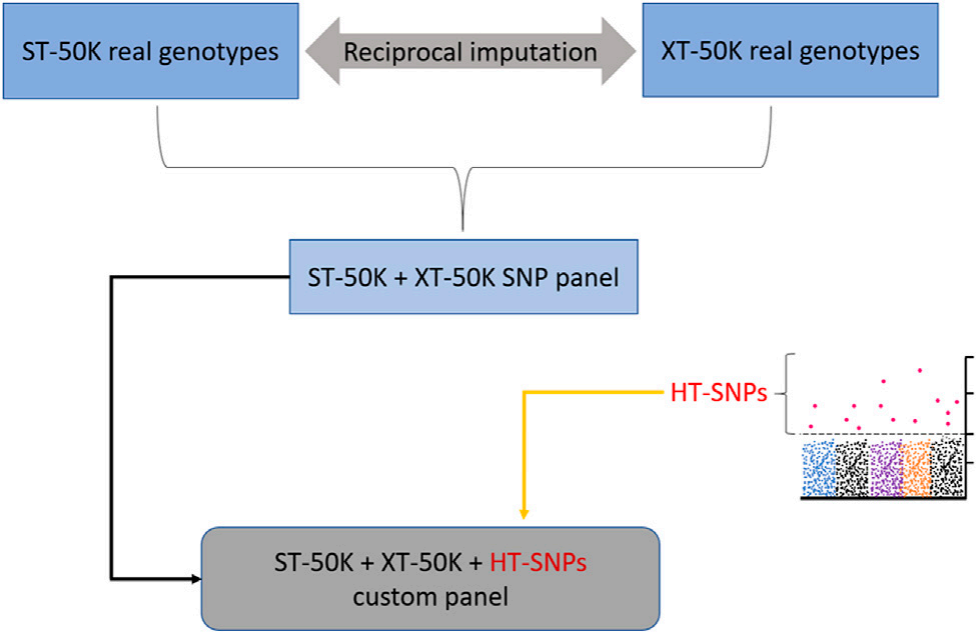
Incorporating a pre-selected “refined” set of heat-tolerance SNPs (HT-SNPs) into the combined set of real genotypes from standard-industry 50 k (ST-50K) and XT-50K SNP panel developed recently by Xiang et al. (2021).

An alternative way by which the industry can benefit from the prioritized heat tolerance variants is to impute them in the “imputation reference” set of animals, such that the SNPs used for routine imputation comprise both the real genotypes and imputed sequence variants for heat tolerance instead of only the real genotypes. While this could enable a faster and cost-effective way of utilizing prioritized heat tolerance variants in the industry, the fact that some imputed variants in the “imputation reference” set are associated with an imputation error may be less appealing to the breeders. In addition, most candidate causal variants for heat tolerance are associated with low minor allele frequencies (i.e., they are rare variants), meaning that they are likely to be imputed with large error (Cheruiyot et al., 2022). However, stringent imputation quality checks can be applied to retain high-quality candidate causal variants for this trait in the “imputation reference” set. There is a proposal in Australia to build an “imputation reference” set of animals that combines real genotype variants for the standard 50k SNP (ST-50K) and XT-50K array (illustrated in Figure 9). The latter panel (XT-50K) was recently developed by Xiang et al. (2021). The new imputation panel (ST-50K + XT-50K) will contain imputed variants from XT-50K that are missing in the ST-50K and vice versa. The new combined panel may provide a convenient way to augment a “refined” set of prioritized heat tolerance variants for routine genomic evaluation in the industry. Therefore, we recommend future tests to see added benefits of integrating a heat tolerance “refined” set of variants into the new combined imputation panel (ST-50K + XT-50K; Figure 9).

### Using Pre-Selected Markers to Design Special SNP Panels

With the increasing availability of whole-genome sequence data in recent years, there has been an evolving trend toward screening causal mutations or variants in high LD with causative mutations for multiple traits and using them to design customized SNP panels for driving genetic improvements for traits in livestock (Liu et al., 2021; Xiang et al., 2021). As noted earlier, Xiang et al. (2021) developed a custom SNP array (called “XT-50K” array) that includes potential causative mutations discovered from dairy cattle across 34 traits, representing milk production, fertility, type, and management. Since heat tolerance traits were not part of the 34 traits analyzed, we recommend that the pre-selected “refined” core set of variants be incorporated in the XT-50K SNP panel in the future and other custom panels used in the industry. This can enable simultaneous improvement of heat tolerance with other traits contributing to farm profitability (i.e., “win for all”). Also, it can help circumvent the possible issues related to low imputation accuracy on genomic predictions if the pre-selected variants are imputed in the industry 50k SNP array since heat tolerance variants are characterized by low minor allele frequency (as noted above). Furthermore, as demonstrated by Khansefid et al. (2020), we expect sustained genomic predictions over many generations for heat tolerance when using the XT-50K array since the prioritized markers are closer to the actual causal mutations. These researchers found that using the XT-50K array yields a consistent and superior accuracy of predictions in crossbred cows than the standard 50K or HD SNP panels—crossbred cows represent “more distant relationships or many generations.”

Also, Cheruiyot et al. (2022) found that using a multi-breed reference set (Holsteins + Jersey bulls) in which pre-selected variants were from only Holsteins yielded a consistent increase in the accuracies for most prediction scenarios. Also, the bias of predictions (calculated as the regression coefficient of the slope phenotypes on the GEBV in the validation sets) decreases (closer to 1) when using a multi-breed compared to a single-breed reference set. These findings emphasize the importance of having a large reference population that represents multiple breeds for training prediction equations for heat tolerance. This is in line with the study by Khansefid et al. (2020), who found that equalizing breeds in the reference set instead of the Holstein-dominated set increases the accuracy and reduces the bias of predictions. Also, van den Berg et al. (2020) found that the reliability of genomic prediction highly depends on the design of the reference set. These authors reported that including a few closely related Holsteins in the reference set (instead of just increasing the size of Holsteins) increased the reliabilities of prediction in the Australian Red dairy cattle. Work is currently underway in Australia to enlarge the size of the reference population through dedicated genotyping of cows with high-quality phenotypes under a project called the Ginfo (Pryce et al., 2018). In line with this, we recommend testing the added benefits of using the updated reference set in addition to considering the “refined set” of prioritized sequence variants.

If the objective is to obtain a “refined” set of SNPs that are beneficial across breeds, then it would be necessary to further prune pre-selected variants for their effect direction and the LD. In line with the abovementioned discussion, variants showing the same direction of effects across Holsteins and Jerseys can be prioritized and included in the XT-50K chip. More importantly, it would be crucial to do further rigorous tests to ensure that the selected “refined SNP set” yields added benefits when they are included in the XT-50K array. Since various biological mechanisms contribute to variations in heat tolerance (morphological, physiological, and behavioral), it is imperative to continue searching for candidate causal variants underlying this trait for inclusion in the custom SNP arrays. Ultimately, this may allow a better understanding of the biology of heat tolerance and facilitate rapid genetic progress for this trait while maintaining milk productivity in farm animals.

While including a “refined SNP set” of variants for heat tolerance in the custom panel (XT-50K) could be the most ideal strategy for the industry to benefit from these markers, we would still expect to see a lag of time, say several years, before this is implemented. This is because re-designing and optimizing such fixed custom arrays is time-consuming and costly, requiring the re-assembly of an “imputation reference” set of animals for routine genomic evaluations. In addition, combining SNPs for heat tolerance with SNPs for other traits (based on meta-analysis) when re-designing such custom chips is likely to “dilute” or diminish the effects of major SNPs for heat tolerance. Furthermore, increasing the number of SNPs in the custom SNP chips (i.e., adding heat tolerance markers) also increases the genotyping costs, which may impede uptake by breeders. Therefore, alternative and more suitable options need to be sought, taking advantage of emerging genotyping technologies, which we discuss next.

### Capitalize on the New Sequencing Technologies to Speed up the Uptake of Prioritized Variants for Heat Tolerance in the Industry

The next-generation sequencing technology is rapidly evolving, thus, increasing efficiency and reducing genotyping costs. For example, the low-pass or low-coverage genome sequencing [also called “skim sequencing” in which a depth of 1× or less of the genome is sequenced] is emerging as a potentially revolutionary tool for genotyping (1000 Genomes Project Consortium, 2015). Recent work in humans (Martin et al., 2021; Rubinacci et al., 2021) and cattle (Snelling et al., 2020) have shown high (>0.90) concordance of variants in the standard SNP arrays versus imputed genotyped calls for individuals that had been sequenced at low coverage (0.5–1×) based on a haplotype reference panel. This implies that skim sequencing could offer a competitive cost-effective alternative in the foreseeable future, thus, potentially replacing the “gold standard” SNP arrays, which have been popular in the market for over a decade now. In addition, skim sequencing is also intuitive in that it can minimize the inherent ascertainment bias of SNP arrays and allows the detection of rare variants for a trait (Rubinacci et al., 2021). Overall, such technological advancements are intriguing, as they could allow screening of many individuals at an affordable cost to identify heat-tolerant genotypes without the need to continuously re-design customized SNP arrays. However, even as this new development unfolds, it would be crucial, at this point, to test the value of the prioritized candidate causal variants for heat tolerance when they are added to the custom SNP panels, such as the XT-50K array (Xiang et al., 2021).

## Future Research Considerations

Heat-tolerance trait definition: in this article, we proposed a new trait associated with thermoregulation called thermal circulation index (TCI), which quantifies the efficiency of heat dissipation as the breeding objective for heat tolerance. As such, future work is needed to explore the suitability of this trait (its heritability and correlation with other economic traits) as the selection target for heat tolerance. Also, it is important to consider other traits for genetic selection of heat tolerance (fertility, health, conformation, and energy balance) since this trait is highly complex, involving multi-faceted biological processes (physiological, behavioral, and morphological).

One of the main challenges to breeding for resilience is possible negative (undesirable) effects on milk production. One possible way to deal with this issue is to explore other heat-tolerance or resilience indicators that are potentially less correlated with milk production (measures of core body temperature.) or to decompose the correlation between these traits. Also, to better understand the genetic control of heat tolerance, comparative studies within and between species are needed, for example, dairy versus beef breeds or even small ruminants such as goats and sheep.

Suitable model(s) for quantifying heat stress: since most THI models used in the genetic evaluation of heat tolerance in many countries do not consider relevant weather elements, for example, solar radiation, the magnitude of heat on the animals could be underestimated, especially for pasture-based herds in Australia. Therefore, work is needed to propose suitable THI model(s) for different dairy systems.

Refine pre-selected SNP set: while it is encouraging that we can increase the prediction accuracy for heat tolerance by using a small set of pre-selected sequence variants, more research is required to obtain a “refined SNP set” that can be added to the industry SNP panels. Also, a rigorous test is required to ensure that the “refined SNP set” yields added benefits when added to the custom SNP panels.

Key biological pathways for heat-tolerance: specific genes and pathways related to the nervous (neuroactive ligand-receptor interaction and glutamatergic synapse) and metabolic (citrate or Krebs cycle) functions are relevant drivers for heat tolerance in dairy animals, which warrants comprehensive follow-up functional studies, for example, looking at their biochemical profiles under different heat stress conditions.

Resilience indicators for multiple traits: as environments continue to become increasingly variable, it may be logical to consider building a multi-trait model for “general” resilience indicators, which encompasses multiple traits that contribute to farm profitability, including heat tolerance, diseases, parasites, and management.

Capture hybrid vigor or heterosis: the specific allele combination or the percentage of adapted breed genetic background required to benefit from heterosis or hybrid vigor advantage for resilience in crossbreds while optimizing productivity remains unknown, which warrants future investigations. Also, crossbreds carrying the well-known heat tolerance SLICK gene and are ranked high for heat tolerance breeding value (e.g., Australian HT-ABVg) could potentially have superior heat tolerance ability and productivity, which requires future interrogations to confirm this hypothesis.

Larger sample size and international collaborations: evidence point to a highly polygenic nature of heat tolerance characterized by many causal variants with small effects. As such, a large dataset with tens of thousands of individuals, such as those typically used in humans (Wood et al., 2014), would be required in future work to detect causal variants with very small effects and the effects of rare causal variants. Sharing data *via* international collaborations is a critical avenue to achieving the large sample size needed for mapping and validating heat-tolerant causal variants. Alternatively, a more feasible approach is to perform a meta-analysis of GWAS results on heat tolerance traits from different countries, for example, meta-GWAS for cattle stature (Bouwman et al., 2018).

## Conclusion

In this review, we have discussed novel discoveries applied to the Australian dairy industry that increase our knowledge of the genetic basis and biology of thermal stress, which may open new avenues for minimizing the effects of heat stress in animals, considering escalating warming climate worldwide. There is substantial genetic variation for heat tolerance (i.e., G × E) among dairy cattle, which producers can benefit from selecting animals that can perform optimally in different environments. For example, if producers are keen on breeding for robustness or resilience to heat, then animals that show consistent performance across different heat stress conditions are ideal for selection. However, dairy animals are becoming more sensitive to environmental changes (i.e., G × E is increasing), in part, because of more emphasis on milk production over the past years and the warming climate. This is a warning signal to the global dairy industry to ensure that G × E is routinely monitored to prevent future economic losses as the climate gets warmer.

Specific candidate causal variants and genes related to the nervous (neuroactive ligand-receptor interaction and glutamatergic synapse) and metabolic functions (citrate cycle) contribute to heat tolerance in animals. It is encouraging that the candidate variants for heat tolerance can be used to increase the prediction accuracy for this trait when they are added to the lower-density industry SNP panels (e.g., 50k array). We discussed several opportunities for which the industry can leverage genetic information to breed animals that can maintain production under hot conditions. Overall, the genetic tools offer promising and long-term prospects for improving resilience aspects in animals, which is crucial in addressing the double challenge of increasing animal production, even more, to feed a growing population while coping with the effects of rising global temperatures and ever-changing production environments.
